# Training programme in gasless laparoscopy for rural surgeons of India (TARGET study) - Observational feasibility study

**DOI:** 10.1016/j.ijso.2021.100399

**Published:** 2021-09

**Authors:** N. Aruparayil, J. Gnanaraj, S. Maiti, M. Chauhan, A. Quyn, A. Mishra, L. Bains, G. Mathew, C. Harris, B. Cundill, A. Fellows, K. Gordon, B. Dawkins, B. Shinkins, J. Brown, D. Jayne

**Affiliations:** a Leeds Institute of Medical Research at St. James's, University of Leeds, UK; b Karunya University, Coimbatore, India; c Kolkata Medical College, Kolkata, India; d Department of Electronic and Electrical Engineering, University of Leeds, UK; e Maulana Azad Medical College, New Delhi, India; f Shanti Bhavan Medical Center, Biru, Jharkhand, India; g Leeds Institute of Clinical Trials Research, University of Leeds, UK; h Academic Unit of Economics, Leeds Institute of Health Science, University of Leeds, UK

**Keywords:** Gasless laparoscopy, Laparoscopic training, LMIC, FLS, OSATS, GOALS

## Abstract

**Background:**

Benefits of laparoscopic surgery are well recognised but uptake in rural settings of low- and middle-income countries is limited due to implementation barriers. Gasless laparoscopy has been proposed as an alternative but requires a trained rural surgical workforce to upscale. This study evaluates a feasibility of implementing a structured laparoscopic training programme for rural surgeons of North-East India.

**Methods:**

A 3-day training programme was held at Kolkata Medical College in March 2019. Laparoscopic knowledge and Fundamentals of Laparoscopic Skills (FLS) were assessed pre and post simulation training using multiple choice questions and the McGill Inanimate System for Training and Evaluation of Laparoscopic Skills (MISTELS), respectively. Competency with an abdominal lift device was assessed using the Objective Structured Assessment of Technical Skills (OSATS) and live operating performance via the Global Operative Assessment of Laparoscopic Skills (GOALS) scores during live surgery. Costs of the training programme and qualitative feedback were evaluated.

**Results:**

Seven rural surgeons participated. There was an improvement in knowledge acquisition (mean difference in MCQ score 5.57 (SD = 4.47)). The overall normalised mean MISTELS score for the FLS tasks improved from 386.02 (SD 110.52) pre-to 524.40 (SD 94.98) post-training (p = 0.09). Mean OSATS score was 22.4 out of 35 (SD 3.31) indicating competency with the abdominal lift device whilst a mean GOALS score of 16.42 out of 25 (SD 2.07) indicates proficiency in performing diagnostic laparoscopy using the gasless technique during live operating. Costs of the course were estimated at 354 USD for trainees and 461 USD for trainers.

**Conclusion:**

Structured training programme in gasless laparoscopy improves overall knowledge and skills acquisition in laparoscopic surgery for rural surgeons of North-East India. It is feasible to deliver a training programme in gasless laparoscopy for rural surgeons. Larger studies are needed to assess the benefits for wider adoption in a similar context.

## Highlights

• Structured training in gasless laparoscopy is feasible for rural surgeons.
• Demonstrates overall improvement in knowledge and skill acquisition.
• The training environment is an essential component for rural surgeons.
• Lower costs support wider adoption in LMIC.

## Abbreviations

MISTELS: McGill Inanimate System for Training and Evaluation of Laparoscopic Skills
OSATS: Objective Structured Assessment of Technical Skills
GOALS: Global Operative Assessment of Laparoscopic Skills
LMICs: Low and Middle-Income Countries
HICs: High Income Countries
MCQs: Multiple-Choice Questions
FLS: Fundamentals of Laparoscopic Skills
ASA: American Society of Anaesthesiologist
INR: Indian rupees
USD: United States Dollar
ICC: Intraclass Correlation Coefficient

## 1 Introduction

The benefits of laparoscopic surgery are well recognised in High Income Countries (HICs), but uptake in rural settings of low and middle-income countries (LMICs) has been limited [1]. This creates an inequality gap, with patients undergoing laparoscopy surgery in HICs benefiting from lower infection rates [2,3] and better outcomes [4] compared to those undergoing open surgery. The main challenges to the introduction of laparoscopy in LMICs are the limited carbon dioxide supply for insufflation, cost and access to laparoscopic equipment, limited anaesthetic cover, and training opportunities [5,6]. Despite these challenges, a few laparoscopic training programmes have been successfully undertaken in Mongolia [7], Botswana [8] and South America [9], proving that it is possible provided resources are available for training and capacity building.

A modified version of laparoscopy, gasless laparoscopy, has been proposed as an alternative to carbon dioxide laparoscopy and overcomes some of the barriers highlighted above [10,11]. It can be performed under spinal anaesthesia for selective abdominal procedures and without the need of carbon dioxide pneumoperitoneum [12]. A recently published phase II randomised controlled trial in India demonstrated no significant difference of gasless as compared to conventional laparoscopy for cholecystectomy and appendicectomy [13]. Other studies in LMICs have shown similarly favourable results [14,15].

In the Disease Control Priorities 3 (DCP 3) document published by the World Bank, it was estimated that by providing essential surgical procedures 1.5 million deaths a year would be averted [16]. Of deaths due to acute abdominal conditions, 9% could have been avoided, and 6.3% of Disability Adjusted Life Years (DALYs) globally could be averted per year if basic surgical care was available [17]. In rural areas of India, surgical services are poorly developed with 90% of the population lacking access to timely surgery [18]. In the North-East of India, surgical provision is 5500 procedures per 100,000 population each year, with around one in eight procedures being potentially amenable to laparoscopic surgery [19,20]. There is an increasing interest amongst rural surgeons in India in gasless laparoscopy as a means to improve surgical outcomes [11,21]. A prerequisite is the need for formal training in the technique to ensure its safe implementation.

So far, no previous training programme has been used to implement gasless laparoscopic surgery in LMICs setting. The aim of the current study was to evaluate a sustainable, structured training programme in gasless laparoscopy for rural surgeons in North-East India, documenting the proficiency gains and the cost of the training programme to inform wider adoption. The main objectives of the study were to evaluate knowledge acquisition, competency in laparoscopic skills, proficiency when using the gasless lift device and to calculate the cost of implementing the training programme to inform scale up.

## 2 Methods

Eight rural surgeons from the North-Eastern Indian states of Assam, Nagaland, Manipur, and Arunachal Pradesh were invited to participate. A three-day didactic and practical training programme was conducted at Kolkata Medical College, a tertiary governmental hospital. Experienced laparoscopic surgeons were invited from secondary and tertiary hospitals in India and the UK to act as laparoscopic trainers with 1 trainee to 1 trainer ratio. Trainers underwent a half day training session on delivery of the study delivered by NA, JG, SM, AQ, DJ. Training included familiarisation with the gasless lift technique, including the device, a detailed introduction to the training course, and the marking schemes. All trainees and trainers completed a Good Clinical Practice online module before participation [22].

**Fig. 1:**
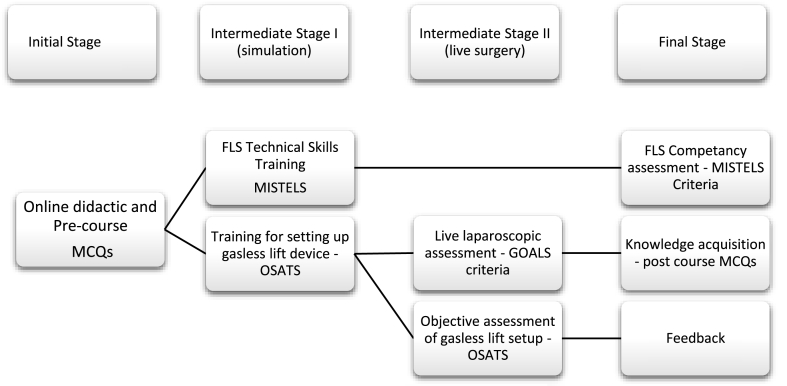
Stages of the TARGET training programme: Initial, Intermediate I & Intermediate II consists of training and assessment. Final stage consists of final results and feedback.

A proficiency-based simulated training curriculum, based on the Fundamentals of Laparoscopic Skills (FLS) [23] and using validated objective tools, was used to assess cognitive and technical skills. The training programme was structured into four phases (Fig. 1): Initial, Intermediate - I, Intermediate –II and Final.

In the initial (pre-training) phase, trainees were provided with online didactic material on conventional laparoscopy, gasless laparoscopy and FLS tasks [24,25]. The online training covered peri-operative considerations for laparoscopic surgery, videos of gasless procedures and instructions on five FLS skills. Pre-training MCQs were used to gain a baseline understanding of the trainees’ knowledge and skills prior to exposure to any of the face-to-face training.

The Intermediate-I simulation phase consisted of training and assessment of technical skills to set up the gasless lift device and FLS laparoscopic skills. The MCQs covered 10 topics relevant to the FLS and gasless laparoscopy curriculum: equipment and energy source, patient considerations, anaesthesia, patient positioning, physiology of pneumoperitoneum/gasless, abdominal access, exposure and examination, biopsy and haemostatic techniques, tissue approximation and site closure, and postoperative care. Each section had 5 questions, with a maximum score of 50. Trainees were then taught to set up the gasless lift device [26] (STAAN, Coimbatore, India), safety features and considerations around pre-, intra- and post-operative considerations, on a silicon based abdominal wall model. Following a demonstration by the principal trainer, technical skills of setting up and insertion of the lift device were assessed by two trainers using the Objective Structured Assessment of Technical Skills (OSATS) [27], where the trainees were marked on seven different aspects of skill with a maximum of five marks per task. Baseline laparoscopic technical skills performance was assessed in five FLS tasks: peg transfer, precision cutting, ligating loop, suture with extracorporeal knot, and suture with intracorporeal knot. Each FLS task was scored using the McGill Inanimate System for Training and Evaluation of Laparoscopic Skills (MISTELS) [28] following online video tutorials. Performance of the five skills tasks were assessed by time and accuracy with penalties assigned for specific errors and lack of precision. Trainees were asked to stop if they reached the maximum time limit without completing the task. FLS training continued with simulation practice of all five FLS tasks with 1:1 trainer-trainee guidance for 6 h.

In Intermediate-II live operating stage, technical skills of setting-up the gasless lift device and laparoscopic skills were assessed. Potentially eligible patients were approached for inclusion in the study by their clinical team. For inclusion, patients had to be > 18 years and requiring elective or acute cholecystectomy, tubal ligation and appendicectomy. Patients with previous multiple surgeries, American Society of Anaesthesiologist (ASA) score of III and above, BMI>25, abdominal trauma, or unable to lie flat were excluded. Informed consent was obtained from all patients. Trainees were expected to perform diagnostic laparoscopy, identifying in a systematic manner the ovaries, fallopian tubes, uterus, appendix, gallbladder, liver and stomach, and performing a 60 cm small bowel assessment. Trainees’ technical performance in setting up the gasless lift device was assessed using OSATS and their live operating performance assessed using the Global Operative Assessment of Laparoscopic Skills (GOALS) [29] by a scrubbed and unscrubbed faculty surgeon. The procedure was completed by a trained surgeon from the host hospital once the assessment was completed.

In the final stage, competency with the FLS tasks and knowledge acquisition was assessed using the same MCQs administered pre-training. General feedback was collected from trainees and faculty using semi-structured interviews and group discussions. The interviews were video recorded and transcribed verbatim. Using a thematic analysis approach, emerging themes related to training experiences were highlighted and summarised in the results. The study has been reported using the STROCSS 2019 Guideline checklist [30], and registered on researchregistry.com - researchregistry6884 - https://www.researchregistry.com/browse-the-registry#home/registrationdetails/60c0d61c374f6f001f59ca22/

### 2.1 Statistical analysis

All analyses were performed using Statistical Analysis System (SAS) version 9.4 (North Carolina, USA) and largely focused on descriptive statistics and confidence interval (CI) estimation due to the small sample size.

For evaluation of the knowledge gained between pre- and post-training, the difference in total scores between the two time points was calculated for each trainee and the overall mean difference and 95% CIs calculated accounting for the paired nature of the data. If a trainee missed a question, they were marked 0 for that question.

The OSATS assessment consists of seven sections, with each marked on a scale from 1 to 5 (1 being the lowest and 5 being the highest). The total score, for the practical session and for each assessor per live operation, was calculated as the summation of each of the individual task scores and ranged from 5 to 35. If a section was not scored the minimum score of 1 was assigned for that section. Higher OSATS scores equates with better proficiency in setting up the device. The average time taken to set up the lift device, and time from skin incision to achieving a satisfactory view, were also summarised.

For each trainee's FLS task, a timing score was calculated from the cut off time minus time to achieve the required proficiency level for that task pre- and post-simulation training. Reaching or exceeding the maximum time results in a score of 0 for that task. For each task the cut off time and penalty score are calculated [31]. A total MISTELS score was derived by subtracting any penalty score from the timing score. Each task score was then normalised by dividing the trainees total score for a task by the best total score achieved by the most qualified trainee, defined by the trainee who had performed the highest number of laparoscopic procedures (trainee 4) and multiplying that number by 100 [28,31]. An overall total score was calculated by summing the normalised total scores from each of the 5 tasks. A higher MISTELS score indicates greater proficiency. The mean and standard deviation of the total normalised scores were calculated for each task pre- and post-simulation training, together with the mean overall score across all five tasks. The Wilcoxon-signed rank test was used to evaluate differences between the pre- and post-simulation training scores as these were chosen a priori to be the most important outcome measure.

The GOALS assessment is made up of 5 sections, each section being scored on a scale from 1 to 5, where 1 is the lowest and 5 is the highest. A total score for each trainee, by live operation and assessor, was derived by summation of the individual section scores, and ranged from 5 to 25. If a trainee was not marked for a section, they were given the minimum score of 1 for that section. Higher GOALS scores equate with better proficiency in laparoscopic skills. Agreement of the scores between the scrubbed and unscrubbed surgeon was assessed using the intraclass correlation coefficient (ICC) and corresponding 95% CI and visualised using Bland-Altmann plots.

### 2.2 Cost analysis

Costs to implement the training programme were monitored throughout the TARGET study and are presented here to inform wider implementation and scale-up. Costs associated with trainer and trainee time to undertake the programme were calculated using a human capital approach by combining time to attend with salary information. Where cover for time out of work had to be arranged, the associated cost was estimated and summarised using a similar approach. Costs associated with delivering the training programme from the training provider perspective were also estimated. These costs included the hire of the training centre, obtaining training materials (including online training materials, simulation trainers etc.), reimbursing trainers for their time and surgical equipment. Costs were estimated in rupees (INR) or pounds sterling (£) in the first instance, depending on the cost information available, but were converted to US dollars (USD) using the conversion rate 1 INR = £0.011 = 0.0146 USD, as of March 2019.

## 3 Results

### 3.1 Trainee characteristics

A total of seven trainees attended the course. The eighth trainee did not attend the course as he was unable to find a cover for hospital duties. The characteristics of the trainees are shown in Table 1. Median (range) years of surgical experience was 7 (3–15) years, with between one- and seven-years’ experience in laparoscopic surgery. The average experience for 6 surgeons was around 20 laparoscopic procedures a year. Most of these procedures had been performed in private practice by three surgeons, as local government hospitals lack laparoscopic facilities. Only one trainee had previously attended a laparoscopic surgical skills course with laparoscopic simulation. None of the trainees had prior experience in performing gasless laparoscopic surgery but four had previous experience of assisting with them.

**Table 1:** Experience of participating trainee surgeons.

	Trainee ID							Total
Characteristic	1	2	4	5	6	7	8	Mean (SD) Median (Range)
Surgical experience (years)	10	7	7	7	3	7	15	7 (3–15)
Number of open surgical courses attended	1	0	0	0	0	0	1	0 (0–1)
Number of previous laparoscopic surgical skills courses attended	0	0	0	0	3	0	0	0 (0–3)
**Previous experience in laparoscopic surgery**								
Number of years’ experience	6	7	7	7	3	7	1	5.43 (2.44)
Number of surgeries performed	60	100	300	150	10	70	N/K	115 (101.73)
**Previous experience in gasless surgery**								
Number of years' experience	4	5	–	0	0	0	2	1.83 (2.23)
Number of gasless surgeries performed	0	0	–	–	–	–	–	0 (0)
Previous training with laparoscopic simulators	0	0	0	0	1	0	0	0 (0–1)

### 3.2 Trainer characteristics

The characteristics of the eight trainers are displayed in table 1 supplement. The median (range) years of surgical experience was 20.75 (7–38) years, with 14.13 (7–25) years of laparoscopic experience. Four of the trainers had some expertise in gasless surgery, with an average of 2.5 (0–5) years. All 8 trainers had previous experience of training surgeons with an average of 11.25 (3–30) years’ experience.

### 3.3 Delivery of the training programme

Only 4 (57.1%) trainees completed the pre-training course webinar and on-line pre-course FLS training material. Of these four, two (50%) also completed the pre-course online gasless training material. All trainees completed online FLS and gasless training didactic modules during the 3-day training programme before undertaking the final MCQ assessment. Trainees received one to one simulation training for around 6 h.

### 3.4 Effects of the training programme on knowledge acquisition

All seven of the trainees completed the pre- and post-training MCQs. Pre-training MCQ scores ranged from 24 to 41, with a mean of 30.14 (SD 7.08). The post-training MCQ scores ranged from 27 to 45, with a mean score of 35.71 (SD 5.71) (Fig. 1 supplement), an average increase of 5.57 (95% CI 1.44–9.7). There was no suggestion that performance was associated with qualifications or previous surgical experience.

### 3.5 Effect of the training programme on setting up the gasless lift device (OSATS score)

Five trainees (71.4%) completed the practical training setting up the gasless lift device on a silicon model of the abdominal wall. OSATS scores ranged from 18 to 34 out of 35 with a similar range of scores overall by the two assessors but some variation between assessors per trainee (table 2 supplement). Live operating OSATS scores ranged from 18 to 29, with a mean score of 22.4 (SD 3.31) and 24.2 (SD 3.30) as assessed by the operating and observing assessors, respectively (Table 2). The time to set up the gasless lift device varied from 36 s to 5 min 30 s, with an average of 1 min 35 s (SD 1 min 46 s). The average time from surgical incision to gaining a satisfactory laparoscopic view was 6 min 54 s (SD 1 min 25 s). Trainees 1 and 4 operated on the same patient and the timings for the second trainee (trainee 4) may have been influenced by the fact that an abdominal incision had already been made.

**Table 2:** OSATS score for live operating. Maximum OSATS score per attempt = 35. Time from skin incision to achieving satisfactory view does not include the time to set-up lift device.

Trainee code	Patient code	OSATS score – scrubbed surgeon (assessor 1)	OSATS score – un-scrubbed surgeon (assessor 2)	Time to set-up lift device	Time from skin incision to achieving satisfactory view
1	901	26	26	1 min 30 secs	5 min 50 secs
2	903	20	20	1 min 11 secs	5 min 3 secs
4	901	23	26	5 min 30 secs	6 min 0 secs
5	904	18	29	0 min 36 secs	9 min 9 secs
6	906	27	24	0 min 38 secs	7 min 49 secs
7	905	20	25	1 min 0 secs	7 min 49 secs
8	907	23	20	0 min 38 secs	6 min 40 secs
**Total** **Mean (SD)**		22.43 (3.31)	24.29 (3.30)	1 min 35 secs (1 min 46 secs)	6 min 54 secs (1 min 25 secs)

### 3.6 Effect of the training programme on trainee competency (MISTELS score)

Table 3 represents the normalised scores for each of the 5 tasks and the overall scores for both pre- and post-simulation training. The overall normalised score total increased from an average score of 386.02 (SD = 110.52) to 524.40 (SD = 94.98) post-training (p = 0.09). For individual tasks, an overall improvement was observed for each task, except for peg transfer which showed a slight decrease due to the process of normalisation such that trainee 4 showed one of the greatest improvements from pre-training peg transfer performance and therefore most of the other trainees appear to have performed worse. Although there was a statistically significant improvement in the overall mean extracorporeal suturing scores (pre = 12.86 (34.02) vs post = 109.74 (20.98); p = 0.03) this should be interpreted with caution due to the small sample size. Figs. 2 and 3 in the supplement illustrate individual trainee scores for the five tasks and overall score between pre- and post-training.

**Table 3:** Normalised MISTELS score pre and post simulation training on FLS - Trainee 2 Post simulation Extracorporeal knot task had faulty equipment and therefore a total score could not be assigned.

Trainee code	Pre-training Overall FLS scores	Post-training Overall FLS scores	P-value
1	424	495	–
2	253	–	–
4ꝉ	400	500	–
5	409	611	–
6	535	643	–
7	456	376	–
8	224	522	–
(Mean ± SD)	386.02 ± 110.52	524.4 ± 94.98	0.09

**Tasks**	**Pre-training (Mean ± SD)**	**Post-training (Mean ± SD)**	
Peg transfer	162.07 ± 78.39	116.11 ± 21.29	0.22
Circle cutting	79.71 ± 42.76	113.23 ± 24.99	0.22
Ligating loop	74.17 ± 46.24	109.17 ± 23.89	0.16
Extracorporeal suturing	12.86 ± 34.02	109.74 ± 20.98	0.03
Intracorporeal suturing	57.21 ± 57.08	65.02 ± 44.35	1

∗ P-value for Wilcoxon-signed rank test between pre and post-tests. ꝉ Trainee 4's raw scores were used to normalise the scores since they are the trainee who has performed the most laparoscopic procedures. The scores to normalise were for pre; Pegs = 58, Circle = 98, Loop = 73, Extra Knot = 0, Intra Knot = 200, then for post; Pegs = 130, Circle = 146, Loop = 109, Extra Knot = 195, Intra Knot = 390.

### 3.7 Characteristics of the patient population

Seven patients were part of the live operating assessment, and their characteristics are shown in Table 3 supplement. Three different surgeries were performed: four tubal ligations; 2 cholecystectomies; 1 appendicectomy. Six of the patients had spinal anaesthetic whilst the seventh had a general anaesthetic as he was unable to tolerate spinal anaesthesia. Four patients had minor intra-abdominal adhesions. There was no intra- or post-operative complications or mortality.

### 3.8 Effect of the training programme on operative laparoscopic skills (GOALS score)

GOALS scores ranged from 13 to 19 out of 25 with a mean of 16.42 (SD 2.07) and 16.29 (SD 2.06) as assessed by the scrubbed and un-scrubbed surgeons (assessors), respectively. The mean time to perform diagnostic laparoscopy was 7 min 10 s (SD 5 min 46 s) (Table 4). Agreement on scoring between the two assessors is demonstrated in the Bland-Altman plot (Fig. 4 supplement).

**Table 4:** GOALS score. Maximum GOALS score per attempt = 25. The diagnostic laparoscopy was performed twice on patient 901.

Trainee code	Patient code	GOALS score - scrubbed surgeon (Assessor 1)	GOALS score - un-scrubbed surgeon (Assessor 2)	GOALS timing
1	901	18	19	2 min 1 secs
2	903	17	15	6 min 33 secs
4	901	16	13	7 min 5 secs
5	904	14	17	5 min 7 secs
6	906	20	17	19 min 0 secs
7	905	15	18	1 min 58 secs
8	907	15	15	8 min 25 secs
**Total** **Mean (SD)**		16.42 (2.07)	16.29 (2.06)	7 min 10 secs (5 min 46 secs)

### 3.9 Cost analysis

Estimated costs associated with the training programme are presented in Table 5. Costs related to trainee and trainer time to attend the course are estimated at 354 USD and 461 USD, respectively. These represent the costs that trainees and trainers might incur to attend the training programme. In the case of trainers, this cost may serve as a lower bound to the fee that organisers may expect to pay trainers for their participation for 3 days. Other unit costs associated with implementing the training programme are presented in Table 5. These may serve as a guide to the costs associated with wider scale-up of this training programme.

**Table 5:** TARGET training programme costs.

Item	Cost (USD)	Source	Details
*Trainee time*			
Estimated cost to attend per trainee	236.00	TARGET study	Based on median 5 days off work and estimated daily rate of INR 3238^a^
Estimated cost of cover per trainee	118.00	TARGET study	Based on 50% of trainees having available cover and estimated daily rate of INR 3238^a^
**Total cost per trainee**	**354.00**		
*Trainer time*			
Estimated cost to attend per trainer	284.00	TARGET study	Based on median 3.5 days off work and estimated daily rate of INR 5550^a^
Estimated cost of cover per trainer	177.00	TARGET study	Based on 62.5% of trainers having available cover and estimated daily rate of INR 5550^a^
**Total cost per trainer**	**461.00**		
*Costs to deliver training programme*			
Hire of training centre	0.00	TARGET study	No formal payment, but provided centre with high end laparoscopic simulator
High end laparoscopic simulator (including needle, scissors & consumables)	2666.00	TARGET study	Advanced training simulator
LapPack training simulator	40.00	TARGET study	Portable training simulator, one provided to each trainee
Trainer fee	1326.00	TARGET study	Fee for trainer participation to facilitate the training and proctorship
Consent form and patient information sheet	0.60	printing and copying charges^3^	2-page consent form and 4-page PIS
Training materials: programme, Questionnaire/testing pack	1.11	printing and copying charges	programme: 1 sheet colour; questionnaires 10 sheets black and white
Attendance certificates	0.12	printing and copying charges	1 sheet single-sided colour
Translation costs^b^	310.00	TARGET study	Consent forms and PIS translated into Hindi and Bengali
Course welcome dinner	32.00	TARGET study	per person

a Daily rates estimated from salary data collected in the TARGET study and assuming 40 h worked per week.

b Translation costs included for reference but would not apply to wider implementation as materials now exist so would not be paid each time.

### 3.10 Qualitative feedback summary

Qualitative feedback was gained from all participants. Emerging themes highlighted the advantages of gasless laparoscopy for not requiring the need of CO_2_ insufflation, and ability to perform selective procedures under spinal anaesthesia. The standardisation of training contrasted previous experiences with both trainees and trainers stating that the TARGET programme taught surgical technique in an organised and structured manner. Most participants thought that gasless laparoscopy was an affordable solution to increase access to minimally invasive surgery in rural settings. The training environment was felt to be important to overall performance and training experience.

## 4 Discussion

Gasless laparoscopy is proposed as an alternative to carbon dioxide laparoscopy in low resource settings, where no formal training and resources exist to ensure safe implementation and uptake. We demonstrate the feasibility and cost-analysis of conducting a training programme in gasless laparoscopy for rural surgeons in NE India. This is the first time that such a training programme has been conducted in a LMIC.

Using validated simulation training and objective assessment tools we have shown an observed overall improvement in knowledge, laparoscopic skills, and ability to safely use an abdominal lift device for gasless laparoscopy. Only the primary outcome was assessed for statistical significance due to the small sample size, however the programme sets a foundation to conduct further studies with a larger cohort of rural surgeons.

Provision of free online didactic material enabled the participants to enhance their knowledge on the basics of laparoscopic surgery and training methods [25,32]. One to one training allowed continuous development of knowledge and skill acquisition. During FLS simulation, the overall normalised MISTELS score deemed to pass the skills component of the programme has to be more than 270 [31]. Most trainees surpassed the 270 cut-offs in the final training assessment and ‘passed’ the FLS criteria for proficiency in a simulated environment. The greatest improvement observed across all trainees was in extracorporeal knot tying skill, with the smallest improvement in intracorporeal suturing. The latter is an important skill which requires bimanual dexterity and more practice time. Tang and colleagues reported the benefits of early exposure to intracorporeal suturing if introduced early in the surgical training [33].

The averages of the overall normalised FLS scores increased over the 3 days training programme with less variation in inter-trainee scores as evidence by the lower standard deviation. Similar improvement in post-test scores were noted in the FLS training programmes carried out in Botswana [34] and Tanzania [35]. The differences in scores observed in other studies may be due to operative experience, FLS standardisation and variation in the normalisation method.

OSATS scores for the live operations were similar or slightly lower than the simulation assessment scores. Lower OSATS scores could be due to limited time and no additional training opportunity between the simulation and live operations. The average time to set up the gasless lift device was 1 min 35 s, and time from making the incision and gain a satisfactory view was 6 min 54 s. Randomised controlled trials on gasless surgery have recorded similar setup times in the range of 5–12 min [13,14]. Further device modifications in the usability of the gasless lift device and proficiency may improve the OSATS scores and reduce the setup times further [36].

The average time to safely perform diagnostic laparoscopy was 7 min. GOALS scores from the TARGET programme were comparable to senior residents performing conventional cholecystectomy in other laparoscopic training programmes [29,37]. The results are likely to improve as training becomes more standardised and embedded in rural surgical training. The procedures’ complexity will also affect the scores and learning curve but are likely to get better with training and experience. Patients, types of procedures and anaesthesia need to be carefully selected before the training programme to ensure safety in a controlled training environment.

The costs associated with the TARGET training programme were relatively low and could be reduced further to make it more sustainable. A randomised control study suggested that low-end laparoscopic simulators and consumables are sufficient to achieve FLS proficiency and reduce costs [38]. The TARGET training programme used simulators and consumables, which are affordable for the local context. As the TARGET training programme was implemented in a study setting, the costs may not represent costs in other contexts. However, they are a guide to the costs that may need to be considered in the implementation and scale up of similar training programmes. Other costs that may need to be considered, but are not presented here, include costs associated with travel, accommodation, and meals/refreshments. Costs related to live operating and theatre use were not included in the costs and deemed part of the patient's free health care package provided by the local government. Organisers delivering the training programme at scale may need to consider local budgets to decide the level of provision available for these items.

The local buy-in from an experienced faculty and professional organisation, such as the Association of Rural Surgeons of India, is important for capacity building and sustainable training in a rural health system [13,39]. Training in the gasless technique was continued in the form of proctorship for the rural surgeons at their own hospitals. This led to the setting up of a prospective registry for gasless procedures to aid safe implementation.

### 4.1 Limitations

Several limitations of this study can be addressed in the future. A small number of trainees participated in the training programme, which limits the ability to draw concrete conclusions about its wider scalability. Due to logistics and time constraints, the entire course was limited to three days only. During simulation training phase, rural surgeons did not have enough time to perform the exact number of repetitions to achieve proficiency level for each FLS task before moving to next. Hence, immediate post-training assessment after practice sessions was not conducted and trainees were assessed only in the final stage of the training programme. However, rural surgeons received around 6 h of one-to-one training with the faculty which was more appropriate for the context of the study. FLS recommends an average of 10 h of practice for complete novices with an average of 6–14 h. Advanced trainees may require a shorter training time. Longer time for simulation practice after initial training is likely to improve scores and performance. Extended training with a greater number of participants could be facilitated with remote proctorship.

Apart from two independent assessors, a third external assessor was initially planned for the GOALS videos assessment but was not feasible due to suboptimal quality of the video recordings and technical challenges during the training programme. Two types of simulators were used for practice sessions: the Pyxus HD (INOVUS, UK) is a commercially available laparoscopic simulator, and the LapPack is a low-cost alternative. The Pyxus HD (INOVUS, UK) simulator was used for pre and post training simulation assessment in this study.

Several local faculty members were introduced to MISTELS, OSATS and GOALS assessment for the first time. This may have led to a variation in impact of training across all trainees and led to variability in assessment and agreement of scores. Detailed pre-course webinars and half day introduction to the course was deemed sufficient to equip the trainers. An alternative would have been to use a Training the Trainers course to train our faculty, and an additional day to gain more experience in the training methods could ensure consistency of training for all trainees. However, this was not possible within the constraints of the project. Calculating the scores can be cumbersome, and an online algorithm could help expedite the marking scheme. The trainees were only allowed to perform diagnostic laparoscopy instead of the entire procedure and may not represent all five GOALS components. Future proctorship sessions and courses require assessments on essential surgical procedures such as appendicectomy, cholecystectomy and tubal ligation to ensure skill acquisition, learning curve and proficiency for the gasless laparoscopic technique.

## 5 Conclusion

The TARGET study has demonstrated the feasibility of establishing a context specific training programme in gasless laparoscopy. The course led to improved overall knowledge, skill acquisition in set up of the gasless lift device, and safe performance of diagnostic laparoscopy. Lower costs support wider adoption of sustainable training programmes in similar LMIC settings.

## Ethical approval

Ethical approval was granted from the University of Leeds School of Medicine Research Ethics Committee (MREC 18–062) and from the Kolkata Medical College and Hospital (MC/KOL/IEC/NON-SPON/333/02–2019).

## Sources of funding

This research was funded by the 10.13039/501100000272National Institute for Health Research (NIHR) (16/137/44) using UK aid from the 10.13039/100013986UK Government to support global health research.

The views expressed in this publication are those of the author(s) and not necessarily those of the NIHR or the UK Department of Health and Social Care.

## Author contribution

NA, DJ, JB, JG, SM, AQ, BC, AF, KG, BD, BS, CH - Study concept and design. NA, JG, SM, AM, LB, GM, AQ, DJ, CH, MC - Training delivery and assessments. NA - Qualitative analysis. NA, CH - Data collection. BC, AF, JB - Data analysis. BD, BS - Cost analysis. NA, BC & DJ - Drafted manuscript. ALL - Critical revisions.

## Registration of research studies

The study has been registered on researchregistry.com - researchregistry6884 - https://www.researchregistry.com/browse-the-registry#home/registrationdetails/60c0d61c374f6f001f59ca22/

## Guarantor

Mr Noel Aruparayil.

## Declaration of competing interest

All authors: Nothing to declare.
