# Supplementary material for: Training programme in gasless laparoscopy for rural surgeons of India (TARGET study) - Observational feasibility study
Source: Int J Surg Open. 2021 Sep;35:None. doi: 10.1016/j.ijso.2021.100399 (PMC8480410; doi:10.1016/j.ijso.2021.100399)
Supplement: Multimedia component 1 [file mmc1.docx]

Supplement

|  | **Trainer ID** | | | | | | | | **Total** |
| --- | --- | --- | --- | --- | --- | --- | --- | --- | --- |
| **Characteristic** | **10** | **11** | **16** | **17** | **18** | **19** | **20** | **21** | **Mean (SD)**  **Median (Range)** |
| Sub Speciality | Urology | Paediatric surgery | General surgery | MAS | General surgery | Gastrointestinal surgery | Colorectal | Colorectal |  |
| **Surgical experience (years)** | | | | | | | | | |
| Years of Surgery | 30 | 38 | 16 | 13 | 12 | 35 | 15 | 7 | 20.75 (11.76) |
| Years of laparoscopic surgery | 16 | 15 | 16 | 7 | 12 | 25 | 15 | 7 | 14.13 (5.77) |
| Years of Gasless Surgery | 5 | 2 | 0 | 1 | 2 | 0 | 0 | 0 | 1.25 (1.75) |
| Number of Gasless Surgery performed | 1000 | 5 | 0 | 9 | 30 | 0 | 0 | 0 | 2.5 (0 - 1000) |
| **Trainer experience** | | | | | | | | | |
| Years of Training surgeons | 30 | 8 | 6 | 5 | 8 | 25 | 3 | 5 | 11.25 (10.25) |
| Number of events they have been a trainer for | 12 | 8 | N/K | 2 | 4 | N/K | 10 | 5 | 6.83 (3.82) |

Table 1: Experience of the trainers

| Trainee code | OSATS score – (assessor 1) | OSATS score – un- (assessor 2) |
| --- | --- | --- |
| 1 | 31 | 18 |
| 2 | 20 | 20 |
| 4 | 25 | 24 |
| 5 | - | - |
| 6 | 29 | 34 |
| 7 | - | - |
| 8 | 24 | 21 |

Table 2: OSATS score - gasless lift device set up simulation assessment using silicon abdominal wall.

| **Patient Code** | **BMI** | **ASA** | **Previous abdominal surgeries** | **Type of surgery** | **Anaesthetic** | **Intra-abdominal adhesions** |
| --- | --- | --- | --- | --- | --- | --- |
| 901 | ≤ 20 | I | No | Tubal ligation | Spinal | Minor |
| 902 | 20 - 22 | I | Yes | Cholecystectomy | Spinal | Minor |
| 903 | 23 - 25 | I | No | Tubal ligation | Spinal | No |
| 904 | 20 - 22 | I | No | Cholecystectomy | General | No |
| 905 | 20 - 22 | II | No | Tubal ligation | Spinal | Minor |
| 906 | 20 - 22 | I | No | Tubal ligation | Spinal | Minor |
| 907 | 20 - 22 | I | No | appendectomy | Spinal | N/K |

Table 3: Patient characteristics included in the study. N/K represents not known. Patient 902 was not included in the assessment of gasless set-up and live operating skills due to the complexity of the surgery.

Figure 1: Box plot displaying the spread of MCQ scores during pre and post-training. Horizontal line within the box is the median and the circle is the mean score. The distribution is skewed to the right; hence the mean is more than the median score.


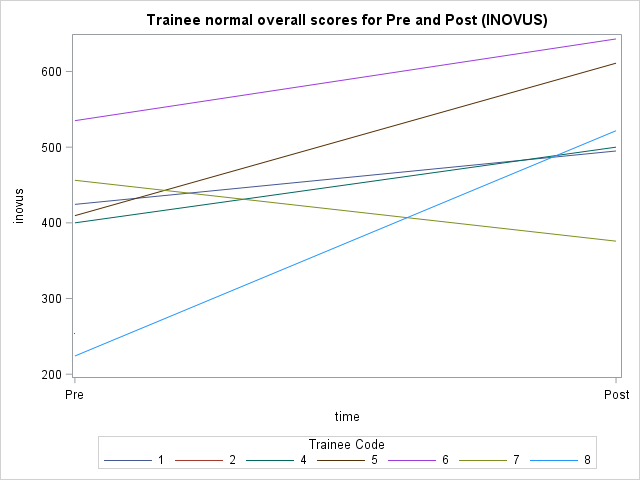


Figure 2: Line graph of trainees pre and post training total FLS scores (data for trainee 2 excluded). Y-axis shows FLS scores for each task.


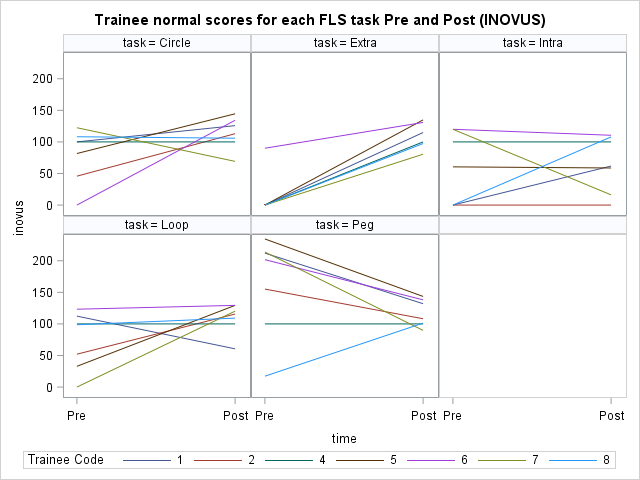


Figure 3 - Line graph of pre and post training scores for each FLS tasks. Y-axis shows FLS scores for each task.


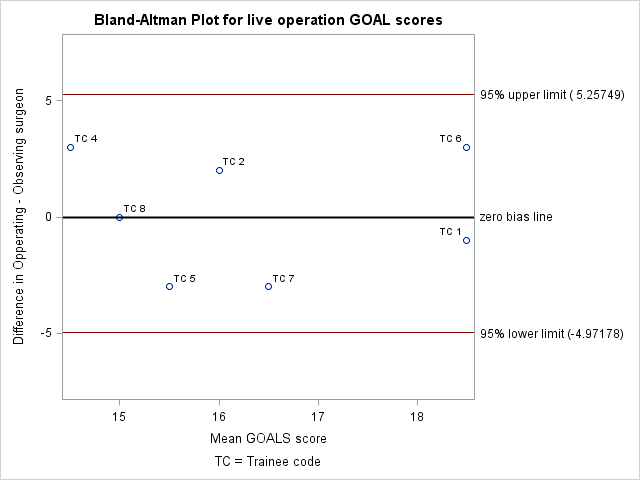


Figure 4: The Bland-Altman plot shows there is reasonable agreement between the two markers as all scores fall between the limits of agreement, although these limits (95% limits -4.97 to 5.26) are wide. The ICC for all operations was -0.019 with the 95% CI -0.64 to 0.62 which shows there is no relationship between the two operating surgeon and observing surgeon scores
